# Researches on the Chemistry of Food

**Published:** 1847-07

**Authors:** 


### ART. XVIII.

*Researches on the Chemistry of Food.* By JUSTUS LIEBIG, M.D., Professor of Chemistry in the University of Giessen. Edited from the Manuscript of the Author, by WILLIAM GREGORY, M.D., Professor of Chemistry in the University of Edinburgh.—*London,* 1847. pp. 176.

WHEN we inform our readers that the preface to this work bears date "Giessen, 1st June, 1847," we feel assured that we have said enough to convince the most unreasonable of them that anything like a review of it (in the sense which we trust we have taught them to understand the word) is utterly out of the question on the present occasion. We regret this circumstance the less, because many parts, we may say, the greater part, admit as yet of no discussion; the volume being almost entirely made up of accounts of processes and results which are stated to have been respectively carried on and obtained in the Giessen Laboratory during the past year. "The present little work," says its author, "contains the analytical details of my investigations on these subjects, [the nature of the organic acid diffused through the muscular system, and that of the other substances contained in that system] which, in accordance with the plan of the Animal Chemistry, could not be introduced into that work." Hence any criticisms that these investigations may evoke at the hands of other chemists will, if necessary, be laid before our readers when the new edition of the Animal Chemistry is completed.

THE FIRST SECTION is devoted, for the most part, to the protein-controversy that has been going on for the last eighteen months between Mulder and Liebig, and as the question must still in a great measure be considered *sub judice,* we will, with a passing expression of our regret that a scientific difference could not have been decided in a less hostile spirit, proceed to the remaining parts of the volume.

SECTION SECOND.—*On the constituents of the juices of flesh.* Our author commences by showing that although Berzelius announced forty years ago that the acid reaction of the juice of flesh depended on the presence of lactic acid, no sufficient proof had ever been given of the real characters by which the acid could be identified, until the present investigations were undertaken; he then alludes to Chevreul's discovery (in the year 1835) of kreatine, to Berzelius's want of success in obtaining it, and to the fact that Wöhler and Schlossberger were enabled to isolate it. These researches constituted the whole of our knowledge in regard to lactic acid and kreatine as ingredients of flesh. As we wish to give an abstract of Liebig's results rather than of his processes, we shall not trouble ourselves or our readers with his improved means of obtaining kreatine. It is at best a complicated and not very easy process, and he himself confesses to "many fruitless attempts." With regard to the physiological relations of this substance, we may observe that it seems to vary considerably in different kinds of flesh. "Of all kinds, the flesh of fowl and that of the martin contain the most, then that of the horse, the fox, the roe-deer, the red-deer and hare, the ox, pig, calf, and finally, that of fishes." (p. 45.)

The amount is greater in wild than in tame animals. The flesh of a fox fed on flesh for two hundred days did not yield so much as the tenth part of the quantity of kreatine obtained from foxes killed in the chase.

"The amount of kreatine in the muscles of an animal stands in an obvious relation to that of fat, or to the causes which determine the deposition of fat. From fat flesh there are often obtained mere traces of kreatine, and always much less than from lean flesh, for the same amount of muscular fibre." (p. 45.)

The following table gives the relative amount of kreatine in the animals specified :

| | | | |
|---|---|---|---|
| 1000 parts of the flesh of | fowl yielded | 3·05 | kreatine. |
| " | the horse | 0·72 | " |
| " | the ox | 0·697 | " |

Liebig has not been able to detect it in the substance of the brain, of the liver, or of the kidneys ; but it is present in abundance in the heart of the ox, so that this organ is especially adapted for its preparation. He describes crystals of pure kreatine as "colourless, perfectly transparent, and of the highest lustre ; they belong to the klinorhombic system, and form groups the character of which is exactly similar to that of sugar of lead. At 212° the crystals become dull and opaque, with loss of water." From four experiments it appeared that the water of crystallization expelled at this temperature averaged 12·17 per cent.

From several analyses it appears that the formula for crystallized kreatine is $C_8N_3H_{11}O_6$, and its atomic weight 149 ; while anhydrous kreatine has for its formula $C_8N_3H_9O_4$, and for its atomic weight 131.

"If we compare the formula of kreatine with that of glycocoll (sugar of gelatine), it appears that crystallized kreatine contains the elements of 2 equiv. glycocoll ($C_8N_2H_8O_6$) + 1 equiv. ammonia ($NH_3$). Kreatine dissolves easily in boiling water, and a solution saturated at 212° forms, on cooling, a mass of small brilliant needles. From a diluted solution it crystallizes very slowly, in somewhat larger crystals, often from two to three lines in length, and one line in thickness, which increase in size for twenty-four hours after cooling, if left in the liquid. 1000 parts of water at 64·4° dissolves 13·44 parts of kreatine, or 1 part of kreatine dissolves in 74·4 parts of water.

"In cold alcohol kreatine is nearly insoluble, 1 part requiring 9410 parts of alcohol for solution. In weaker spirits of wine it is rather more soluble.

"The cold aqueous solution of kreatine possesses, from the small quantity of dissolved matter, a weak, bitter taste, followed by a somewhat acrid sensation in the throat. When the aqueous solution of kreatine contains a trace of foreign organic matter, it decomposes very readily.........

"No quantity, however large, of kreatine can destroy the acid reaction even of the weakest acids, and it possesses no basic characters. It dissolves easily with the aid of heat in barytic water, and crystallizes from it unchanged....... But when boiled with barytic water, kreatine is decomposed, ammonia is disengaged, the liquid becomes turbid, even when the air is entirely excluded, and there is deposited carbonate of baryta in crystalline grains, the quantity of which progressively increases as the boiling is continued.. ......

"The action of strong mineral acids is very remarkable. A solution of kreatine, to which, while cold, hydrochloric acid is added, gives by spontaneous evaporation crystals of unchanged kreatine. But when heated with strong hydrochloric acid, a solution of kreatine no longer yields crystals of that substance. The same result is obtained with sulphuric, phosphoric, and nitric acids. When kreatine is dissolved in one of these acids, and the solution gently evaporated, crystals are obtained, which are very soluble in alcohol, a property not belonging to kreatine. These crystals contain a portion of the acid employed, in a state of combination.

"There is formed in this reaction from kreatine, by a transformation of its elements, caused by contact with strong mineral acids, a new body of totally different properties, a true organic alkali, which I shall call *kreatinine*." (pp. 51-3.)

*Kreatinine, its preparation, properties, &c.* Kreatinine may be obtained in a state of purity from its hydrochlorate or sulphate. The crystals belong to the monoklinometric system, and their measurements have been determined by Kopp. Kreatinine is much more soluble in cold water than kreatine, one part dissolving in 11·5 parts of water at 60°; and in hot water it is much more soluble. It communicates an alkaline reaction to its solution. It dissolves in boiling alcohol, and crystallizes on cooling. 1000 parts of alcohol at 60° dissolve 9·8 parts of kreatinine.

By various reactions it is shown to be a powerful base; in fact, Liebig observes that, "in its chemical character, kreatinine is quite analogous to ammonia."

The conversion of kreatine into kreatinine is shown to depend on the separation of 4 eqs. of water.

$$\text{Kreatine } (C_8N_3H_{11}O_6) = \text{Kreatinine } (C_8N_3H_7O_2) + 4\ HO.$$

"If we compare with the formula for kreatinine that of caffeine (théine), it appears that kreatinine contains the elements of 1 atom of caffeine + 1 atom amide.

"Caffeine is $C_8N_2H_5O_2$: add to this

1 at. amide $N\ H_2$

The sum is $C_8N_3H_7O_2$ = 1 at. kreatinine." (p. 59.)

We now pass from the consideration of the fluid of muscular flesh to that of the urinary secretion. Those of our readers who are conversant with the chemistry of the secretions, must recollect that about three years since a new nitrogenous substance was discovered almost simultaneously in it by Pettenkofer and Heintz. This substance contains the same proportions of carbon and nitrogen as kreatine and kreatinine; in fact, Liebig shows that it is nothing more than a mixture of kreatinine with a little kreatine, which may be easily separated from one another by means of alcohol; one of them (as we have already stated) being easily soluble, the other very sparingly soluble in hot alcohol.

The composition of these two substances, as obtained from urine, was found to be as follows:

| | Kreatine from urine. (anhydrous.) | Kreatinine from urine. |
|---|---|---|
| Carbon | 36·90 | 42·64 |
| Nitrogen | 32·61 | 37·41 |
| Hydrogen | 7·07 | 6·23 |
| Oxygen | 23·42 | 13·72 |
| | 100·00 | 100·00 |

"If we compare these numbers with those obtained by the analysis of kreatine from flesh and the analysis of the kreatinine prepared from it, it is obvious that they are respectively identical, and indeed no difference can be detected in the physical and chemical characters of the two substances from urine and those from flesh." (p. 64.)

During the putrefaction of urine, the kreatine is destroyed by the action of the carbonate of ammonia formed from the urea, while the kreatinine suffers no change.

Liebig regards the urine as not only the most economical but also the most convenient source of these compounds.

In a subsequent portion of the work it is shown that kreatinine is an actual constituent of muscle, or, in other words, that it exists ready formed in flesh, and a simple process for extracting it is given.

We now arrive at the consideration of a new substance, *Sarcosine,* obtained by the action of boiling barytic water on kreatine.

*Sarcosine* crystallizes in right rhombic prisms, acuminated on the ends by surfaces set perpendicular on the obtuser angles of the prism. The crystals are colourless, perfectly transparent, and of considerable size. They are extremely soluble in water, very sparingly soluble in alcohol, and insoluble in ether.

The aqueous solution of sarcosine has no action on vegetable colours, but from the mode in which it combines with acids it evidently acts as a powerful base. Liebig deduces for it the formula $C_6N_1H_7O_4$, which serves to explain its production from kreatine.

"If from the elements of crystallized kreatine we subtract those of sarcosine, there remains a formula exactly identical with that of urea:

| | |
|---|---|
| From 1 eq. kreatine | $= C_8N_3H_{11}O_6$ |
| Deduct 1 eq. sarcosine | $= C_6N\ H_7\ O_4$ |
| There remains 1 eq. urea | $= C_2N_2H_4\ O_2$ |

"It is consequently obvious that, in the decomposition of kreatine by baryta, carbonic acid and ammonia are secondary products derived from the decomposition of urea." (p. 75.)

In further confirmation of this view, Liebig has not only ascertained that a solution of urea in barytic water is resolved by long boiling into carbonate of baryta and ammonia, with the same appearances as those occurring in the production of sarcosine, but further, that urea is present in the liquid when kreatine is boiled with baryta, if examined before the whole of the kreatine is decomposed.

Sarcosine and urea are not the only products of the decomposition of kreatine by baryta; another substance of a feeble acid reaction, but not combined with baryta, was observed; it crystallized in long colourless prisms or scales, very soluble in water and alcohol. The quantity was too small to admit of an ultimate analysis.

*Inosinic acid.* When the liquid from flesh has entirely deposited the crystals of kreatine, and is somewhat further concentrated by evaporation, if alcohol be added to it in small quantities till the whole becomes milky, it deposits in the course of a few days yellowish or white crystals of salts of inosinic acid. The solution of inosinic acid has "a strong acid reaction, and possesses an agreeable taste of the juice of meat." When evaporated, it yields a syrup which after weeks exhibits no signs of crystallization. If this syrup be mixed with alcohol, the thick viscid fluid is changed into a hard, firm, pulverulent mass, of which alcohol dissolves only traces. From a concentrated aqueous solution the acid is precipitated in white amorphous flocculi.

From analyses of its baryta-salt the anhydrous acid appears to be represented by the formula $C_{10}N_2H_6O_{10}$, and if we suppose the baryta replaced by its equivalent of water the formula of inosinic acid will be

$$C_{10}N_2H_7O_{11} = C_{10}N_2H_6O_{10} + HO.$$

"Inosinic acid appears from its composition to belong to the coupled acids. Considered as a hydrate, it contains the elements of acetic acid, oxalic acid, and urea:

| | |
|---|---|
| 1 eq. anhydrous acetic acid . . . . | $C_4 \quad H_3O_3$ |
| 2 eq. anhydrous oxalic acid . . . . | $C_4 \qquad O_6$ |
| 1 eq. urea . . . . . . . . | $C_2\ N_2H_4O_2$ |
| 1 eq. hydrated inosinic acid . . . . | $C_{10}N_2H_7O_{11}$ (p. 84.) |

*Lactic acid* is next shown to be a constituent of flesh, and the best methods of preparing and purifying it are given. The lactates of lime and zinc were prepared from this source, and submitted to ultimate analysis.

In a subsequent part of the work the purposes of lactic acid in the organism are made an object of inquiry. In the first place, it is shown that this acid does not occur in healthy urine, neither is it formed from it during the process of putrefaction; and, secondly, it cannot be detected in that secretion when taken internally as lactate of potash.

"From this it plainly appears that the lactic acid in the organism is employed to support the respiratory process, and the function performed by sugar, starch, and in general all those substances which, in contact with animal matter, are convertible into lactic acid ceases to be an hypothesis. These substances are converted in the blood into lactates, which are destroyed as fast as they are produced, and which only accumulate where the supply of oxygen is less, or where some other attraction is opposed to the agency of that element." (p. 103.)

*The inorganic constituents of the juices of flesh* are then discussed at considerable length. Liebig asserts that the ash of the juice of meat contains only alkaline phosphates and chlorides, there being no carbonates or sulphates present. The soluble salts contain the different modifications of phosphoric acid, which are easily distinguished by their action on nitrate of silver.

"The ashes of the juice of flesh, in the case of the ox, horse, fox, and roe-deer, give with water a strongly alkaline solution, which is precipitated, first white, then yellow, by neutral nitrate of silver; and the mixture, after complete precipitation, is perfectly neutral. This proves that the ashes contain salts of phosphoric acid, with 2 atoms (pyrophosphates), and with 3 atoms (tribasic phosphates) of fixed alkaline base. . . . . . . . The ashes of the juice of the flesh of fowl give a different result. The aqueous solution precipitates nitrate of silver pure white; the ashes, therefore, contain alkaline pyrophosphates; and when they are acted on by nitric acid and again ignited, the soluble portion still precipitates nitrate of silver only white, although an additional quantity of alkali is then added to the phosphate originally present. From this it follows that the juice of the flesh of fowl must contain a certain, though small, quantity of alkaline phosphate, with 1 atom of fixed base (metaphosphate), since, otherwise, after the action of nitric acid on the ashes, a certain quantity of phosphate with 3 atoms of fixed base (tribasic phosphate) must have been produced, and thereby a yellow precipitate must have been formed, to a corresponding extent, in the nitrate of silver.

"The whole amount of alkalies, therefore, present in the juice of the flesh of the ox, horse, fox, and roe-deer is not sufficient to convert the phosphoric acid of the juice entirely into the so-called neutral salt, that is, the salt with 3 atoms of fixed base. In the fowl the whole of the alkali is not even sufficient to convert the phosphoric acid entirely into the salt with 2 atoms of fixed base." (pp. 97-8.)

But the juice of flesh even before all the phosphoric acid has been precipitated by baryta, at a period, therefore, when it can contain no baryta dissolved, acquires an alkaline reaction.

"Hence it follows, that the organic acids present in the juice, the lactic and inosinic acids, &c., taken together, are not in sufficient quantity to form neutral salts with the alkalies contained in it, potash and kreatinine; and this necessarily implies that the acid reaction of the juice of flesh is caused by the presence of acid salts of the alkalies with the three acids—phosphoric, lactic, and inosinic acids. Inosinic acid constitutes too small a part of the juice to allow us to ascribe to it a perceptible share in producing the acid quality of that fluid; and this acidity depends therefore on the presence of acid alkaline lactate and acid alkaline phosphate (phosphate with one atom of alkali), or, in other words, of neutral alkaline lactate and phosphate along with free lactic and phosphoric acids." (pp. 98-9.)

It is thus obvious that we have the condition of an electrical current present. There is an alkaline fluid circulating in the blood-vessels and lymphatics, while the surrounding fluid, that of the flesh, is acid, and the tissue of which the vessels are composed is permeable for the one or the other of these fluids. Liebig considers it "far from improbable that such a current takes a certain share in the vital processes, although its action be not always indicated by proper electrical effects." (p. 104.)

We now come to some very important points regarding the differences of the salts occurring in the juice of flesh, and in blood and lymph: while the former contains phosphate of potash and chloride of potassium, the latter fluids contain phosphate of soda and chloride of sodium. A method is given by which the relative proportions of potash and soda in the juice of flesh and in blood may be determined, and by which the materials for the following table were obtained:

| | | Potash in the blood. | Potash in the flesh. |
|---|---|---|---|
| For 100 parts of soda in the | fowl . . . | 40·8 | 384 |
| ,, | ox . . . | 5·9 | 279 |
| ,, | horse . . | 9·5 | 285 |
| ,, | fox . . . | .. | 214 |
| ,, | pike . . . | .. | 497 |

"It is hardly necessary to state that these numbers only express approximatively the proportions of potash to soda in the flesh, because it is impossible to obtain the juice of the flesh of the ox, horse, and fowl free from blood and lymph, fluids which contain much soda. Had it been possible to obtain the juice of flesh unmixed with blood and lymph, the proportion of potash to soda would have come out much higher, so much so, indeed, that the conclusion that salts of soda form no part of that fluid is not destitute of probability; and if, as is supposed, the lymphatic vessels possess the power of taking up the salts of soda which pass from the capillaries into the substance of the muscles, and returning these salts to the larger blood-vessels, the fact just mentioned admits of a very easy explanation. From the great difference of chemical nature and qualities in the fluids circulating in the different parts of the organism, it follows that there must be a very remarkable difference in the permeability of the parietes of the vessels for these fluids. Were this permeability in all cases the same, there must have been found as much of the salts and potash in the juice of flesh as in the blood; but the blood of the ox and the fowl contains nearly a third of its whole saline contents of chloride of sodium, while hardly a trace of this compound occurs in the juice of flesh. The vessels which secrete milk must stand in a similar relation to the blood-vessels; for in the milk of the cow the salts of potash preponderate very greatly over those of soda, and are present also in much larger quantity than in the saline constituents of blood.

"In some pathological conditions there has been observed,* at points where bones and muscles meet, an accumulation of free lactic and phosphoric acids, which has never been perceived at those points in the normal state. The solution and removal of the phosphate of lime, and therefore the disappearance of the bones, is a consequence of this state. It is not improbable that the cause, or one of the causes, of this separation of acid from the substance of the muscle is this,—that the vessels which contain the fluid of the muscles have undergone a change, whereby they lose the power of retaining within them the acid fluid they contain." (pp. 107-8.)

The next point noticed by Liebig is *the importance of chloride of sodium to the formation of blood.* The constant preponderance of the soda-salts in the blood and the potash-salts in the juice of flesh, justifies the assumption that their presence in these fluids is indispensable. The question then arises what is the source of the soda-salts, especially the phosphate of soda found in the blood? The ox and the horse must obtain their inorganic constituents from the vegetables on which they feed, and analyses have indisputably proved that plants growing at a certain distance from the sea contain no soda, or only traces of that base, while they abound in potash-salts. The answer to the question regarding the source of the phosphate of soda in the blood must be sought for in the investigation of the action of the phosphate of potash (yielded by the food) on chloride of sodium (taken additionally).

"Phosphate of potash with two atoms of potash (tribasic phosphate of potash with two atoms of fixed base, and one atom of water) = $PO_5 \left\{ \begin{matrix} 2\ KO \\ HO \end{matrix} \right\}$, is deliquescent, hardly crystallizable, and has a very feeble *alkaline* reaction.

When we supersaturate phosphoric acid (tribasic) with potash, and evaporate to crystallization, a salt is deposited which has an *acid* relation = $PO_5 \left\{ \begin{matrix} KO \\ 2\ HO \end{matrix} \right\}$. There is no salt which loses half the amount of base it contains so easily as the phosphate of potash. If phosphoric acid be neutralized with potash, and chloride of sodium added to the solution, and the whole left to spontaneous evaporation, a phosphate crystallizes, which contains both potash and soda (the tribasic salt $PO_5 \left\{ \begin{matrix} NaO \\ KO \\ HO \end{matrix} \right\}$) while chloride of potassium is found in the mother liquid.

"It is obvious that phosphate of potash is decomposed when in contact with chloride of sodium; part of the potassium combines with the chlorine, while the sodium replaces it in the phosphate; phosphate of soda being produced.

"In this way we can understand the formation of phosphate of soda in the body of an animal which obtains in its food, along with phosphate of potash or earthy phosphates and salts of potash, no compound of soda except chloride of sodium; and when in inland countries the food does not contain common salt enough to produce the phosphate of soda necessary for the formation of the blood, then more salt must be added to the food." (p. 110-11.)

We regret that our limits will not permit of our entering on the explanation of Liebig's views regarding the uses of the phosphate of soda contained in the blood. We must content ouselves with the simple announce-

* Schmidt, Annalen der Chemie u. Pharmacie, vol. 61, p. 329.

ment of his belief, that the blood owes its power of absorbing and giving off carbonic acid to the presence of this constituent.

The section concludes with some remarks on the relative proportions of lime, magnesia, and alkalies to the juice of flesh, and on the best modes of determining them.

Section third.—*Practical application of the results of the foregoing investigation.* This must be regarded as the first attempt to treat cookery on chemical and philosophical principles. Our author commences with the subject of *boiling.* It is obvious that if flesh is boiled in a sufficient quantity of water and for a sufficient time, the fluid must extract soluble alkaline phosphates, lactates, inosinates, phosphate of magnesia, and slight traces of phosphate of lime; while the boiled flesh contains the insoluble inorganic constituents and the fibrine, fat, &c. In consequence of the importance of the soluble constituents to the organism, it follows "that boiled flesh when eaten without the soup formed in boiling it (the *bouilli* without the *bouillon*) is so much the less adapted for nutrition, the greater the quantity of the water in which it has been boiled, and the longer the duration of the boiling." (p. 123.) The smell and taste of roasted flesh arise from the soluble constituents of the juice, which have undergone a slight change under the influence of the higher temperature; it is also on the soluble matter that the flavour of soup and of the different kinds of meat depend.

The flesh of old animals contains little albumen, but much fibrine; in fact, the quantities of albumen and fibrine seem always to stand in an inverse ratio to one another.

"The tenderness of boiled or roasted meat depends on the quantity of the albumen deposited between the layers and there coagulating; for the contraction or hardening of the fibrinous fibres is thereby to a certain degree prevented. This quality, tenderness, however, also depends on the duration of the boiling, for the albumen also becomes harder by continued boiling without, however, assuming a tough consistence." (p. 125.)

The following is given by Liebig as the best method of boiling meat:

"If the flesh intended to be eaten be introduced into the boiler when the water is in a state of brisk ebullition, and if the boiling be kept up for some minutes, then so much cold water added as to reduce the temperature of the water to 165° or 158°, and the whole kept at this temperature for some hours, all the conditions are united, which give to the flesh the quality best adapted to its use as food." (p. 126.)

If, however, meat is to be boiled for the purpose of making soup, a very different course must be pursued. It should be placed in cold water, which must be *very gradually* brought to the boiling point. In this way there occurs from the commencement of the process an interchange between the juices of the flesh and the external water, which would be altogether impossible according to the directions we have given above for boiling meat, in consequence of the immediate coagulation of the albumen that in that case takes place in the vicinity of the surface, and prevents the penetration of water into the interior of the mass. Liebig proves, experimentally, that boiling water allowed to act for five hours on finely-chopped flesh does not dissolve more than the fifth part of the matters soluble in cold water, even after the albumen has been separated by heating the cold infusion. We cannot refrain from extracting Liebig's prescription for soup.

"When 1℔ of lean beef, free of fat, and separated from the bones, in the finely chopped state in which it is used for beef-sausages or mince-meat, is uniformly mixed with its own weight of cold water, slowly heated to boiling, and the liquid, after boiling briskly for a minute or two, is strained through a towel from the coagulated albumen and the fibrine, now become hard and horny, we obtain an equal weight of the most aromatic soup, of such strength as cannot be obtained, even by boiling for hours, from a piece of flesh. When mixed with salt, and the other usual additions by which soup is usually seasoned, and tinged somewhat darker by means of roasted onions or burnt sugar, it forms the very best soup which can in any way be prepared from 1℔ of flesh." (pp. 131-2.)

By the concentration of such a soup on the water-bath, till it ceases to lose water, we obtain a dark brown, soft mass, of which half an ounce suffices to convert 1lb. of water, with the addition of a little salt, into a strong well-flavoured soup. This is true portable soup, while that of commerce almost invariably consists of mere gelatine.

"Of the true extract nearly 80 per cent. is soluble in alcohol of 85 per cent., while the ordinary tablets of portable soup rarely yield to that menstruum more than 4 or 5 per cent. The presence of kreatine and kreatinine, the latter of which is instantly detected by the addition of chloride of zinc to the alcoholic solution, as well as the nature of the salts left on incineration, which chiefly consist of soluble phosphates, furnish sufficient data for judging of the quality of the true extract of flesh." (pp. 133-4.)

Liebig considers this extract of meat as most valuable for ships and fortresses, and in fact in all cases where fresh meat and vegetables are wanting, and the people are supported on salt meat.

*Salted meat* is not sufficient to preserve the organism in a condition of health, for the brine formed by rubbing the salt into the flesh contains the chief constituents of a concentrated soup or extract, namely, soluble phosphates (especially phosphate of potash), lactic acid, kreatine, and kreatinine. If these substances be not supplied from other quarters, the juices of the flesh cannot be retained in their normal state.

"A change in the quality of the gastric juice, and consequently in that of the products of the digestive process, must be regarded as an inevitable result of the long continued use of salted meat; and if, during digestion, the substances necessary to the transformation of that species of food be taken from other parts of the organism, these parts must lose their normal condition." (pp. 135-6.)

Liebig believes that meat acted on by salt containing chlorides of calcium and magnesium would probably be less injurious than when salted in the ordinary manner.

The only subject remaining for consideration in this section is the *gastric juice*. Lehmann's experiments proving that lactic acid is present in that secretion in dogs are admitted as perfectly decisive; in fact, our author is now as ready to find lactic acid, as he was a very short time ago to disprove its existence in the body.* He regards the gastric juice as similar to the juice of flesh, and he is consequently of opinion that his soup may, perhaps, admit of being employed as a valuable remedy in many cases of dyspepsia, with a view to increasing the activity of the stomach and promoting digestion.

* "In a normal state of health, *no lactic acid* is formed in the stomach." (Liebig's Animal Chemistry, &c., p. 112, 2d edition, 1843.)

The presence of free hydrochloric acid in this fluid is no longer requisite for the carrying out of his theories, and singular to relate, that acid no longer exists there. In the second edition of the 'Animal Chemistry,' published four years since, we are informed that "the presence of free muriatic acid in the gastric juice, first observed by Prout, has been confirmed by all those chemists who have examined that fluid since;" but now (June, 1847),

"when we recollect that lactic and phosphoric acids, at temperatures in which hydrochloric, acetic, and barytic acids are volatilized, are almost fixed, we can explain how it happens that, in many cases, hydrochloric acid, in others, acetic or butyric acid, has been obtained by distilling the gastric juice. Acetates, butyrates, and even chloride of sodium are decomposed by lactic acid, as well as by acid phosphates in these circumstances, and the occurrence of the one or the other of the more volatile acids must vary with the amount of the lactic or phosphoric acid present in the gastric juice, and the amount also of their salts in the same fluid." (p. 139.)

In the preceding pages we have given our readers an abstract of all the more important parts of this volume, which we deem the most valuable that has yet appeared from the pen of its illustrious author, in so far as concerns the application of animal chemistry to the actual practice of medicine.

We have alluded in one or two instances to the differences of opinion, on points of high importance, occurring in the present volume and in the last edition of the 'Animal Chemistry,' and we might have given more illustrations of a similar nature. We have done so with the view of showing that if, from the evidence afforded by his own writings, the first chemist of his age may fall into grievous errors, he should look with some degree of forbearance on those who have the misfortune to differ from him in points of comparatively minor importance. However, the points of controversy between himself and Mulder may be ultimately decided, his warmest admirers must ever regret the unaccommodating and hostile spirit he has exhibited. The following sentence from Mulder's defence* is strong: we do not say that it is altogether true; but the very fact that such opinions are entertained by others ought to make the great professor more cautious and less dogmatical in the expression of his own. "Freedom of scientific opinion has never been understood by Liebig. For years past a tribunal has been established in Giessen, before which Liebig is at the same time accuser, witness, public prosecutor, advocate and judge."

---

### Art. XIX.

* Mulder's Reply to Liebig, p. 6.

